# Supplementary material for: Are carbon emissions trading and green financial instruments synergistic? -Comprehensive quantitative research based on content analysis
Source: PLoS One. 2024 Mar 7;19(3):e0298601. doi: 10.1371/journal.pone.0298601 (PMC10919601; doi:10.1371/journal.pone.0298601)
Supplement: S1 Data — (ZIP) [file pone.0298601.s001.zip › Data/地级市Z_F工作报告.zip!/╝¬┴╓/╝¬┴╓╩╨/└┤╫╘╣1⁄2╓┌║┼í╛┬φ┐╦╩2╛▌═°í┐_2019.docx]

各位代表：

　　我代表市政府向大会报告工作，请予审议，并请政协委员提出意见。

　　一、2018年工作回顾

　　过去的一年，是吉林市振兴发展中极不平凡的一年。面对错综复杂的严峻形势和艰巨繁重的发展任务，我们坚持以习近平新时代中国特色社会主义思想为指引，按照省委、省政府部署，在市委的领导下，紧紧团结和依靠全市干部群众，坚持稳中求进工作总基调，落实高质量发展要求，经济社会保持平稳健康发展。

　　——经济增长稳中有进。地区生产总值增速逐步回升，在上半年增速下降1%的基础上，前三季度增长1.2%，全年预计增长2%左右。固定资产投资增速由负转正，全年预计增长4%以上。社会消费品零售总额逐季增长，全年预计增长4.6%。外贸进出口总额首次突破80亿元，增长21%，高于全省13个百分点。

　　——质量效益稳中向好。一般公共预算收入实现375.5亿元，增长1.8%。地方级财政收入增长4.5%，高于预期1.5个百分点，其中，税收收入增长5.6%，非税收入占地方级收入比重下降0.8个百分点。全市规模工业总产值增长6.9%，增加值预计增长3.8%，其中，百户重点企业产值增长9.1%，实现利润76亿元。单位GDP能耗下降3.5%。

　　——营商环境大幅提升。率先在全省启动“只跑一次”改革，取消各类证明1000余项。推进“证照分离”改革，企业开办时间压缩至3个工作日以内。全市新登记各类市场主体4.6万户，民营企业户数增长17%，民营经济主营业务收入增长7%。

　　——人民生活不断改善。城镇新增就业7.7万人。城镇和农村常住居民人均可支配收入预计分别达到28400元和13750元，分别增长4.5%和5%，高于GDP增速。

　　一年来，主要做了以下工作：

　　（一）现代产业体系加快构建

　　冰雪产业快速发展。认真贯彻习近平总书记“冰天雪地也是金山银山”的重要指示精神，立足我市冰雪资源优势，抢抓2022北京冬奥机遇，着力推进冰雪产业发展。建立工作推进机制，确定年度十个方面40项重点任务，明确部门责任。高标准编制全市冰雪产业发展规划、雪场资源规划和泛北大壶区域冰雪产业发展规划，通过中央电视台、北京冬博会、欧洲阿尔卑斯山地博览会等平台，广泛宣传我市冰雪资源。项目建设取得明显成效，中瑞滑雪小镇项目签订协议，北大壶滑雪度假区新开工重点项目15个，总投资36亿元，增长76%；万科松花湖滑雪度假区新开工项目投资增长85%。亚洲首座全天候标准化越野滑雪训练场——北山四季越野滑雪场建成。推动全民上冰雪，实施“筑梦冰雪相约冬奥”中小学生冰雪运动普及工程，省级冰雪特色校发展到75所，市民及青少年上冰雪分别达到230万和20万人次。北华大学成立全国首家冰雪学院。全年接待国外冰雪考察洽谈团组28个，占全市对外交流团组的40%。新雪季以来，全市规模雪场接待滑雪者人次、旅游收入分别增长44%和34%，我市冰雪产业对外影响力明显提升，产业发展趋势得到国内外高度认可。新兴产业发展壮大。碳纤维步入产业化、规模化发展新阶段，碳谷原丝项目新增产能8000吨，吉林精功一期2000吨大丝束碳纤维实现量产，碳纤维原丝产能、碳丝单线产能位居全国第一，产业产值增长61.7%。医药健康产业保持较快发展，康乃尔原料药试生产，吉尔吉医药健康产业园等项目全面投产。国家级通航产业综合示范区建设步伐加快，吉航民机维修基地建成，金汇全省直升机应急救援基地运营。工业智能化转型初见成效，金洪汽车数字化车间建成，吉林化纤等5条数字化生产线投入使用，万丰奥威轮毂单元智慧工厂被工信部确定为智能制造新模式应用项目。传统产业转型步伐加快。吉化汽油国Ⅵ质量升级烷基化、吉神聚醚多元醇等化工“补链”项目竣工投产。吉林铁合金、吉林炭素正式签订搬迁协议，吉林建龙80万吨冷轧钢项目主体完工。一汽吉林汽车7个新能源新车型通过工信部生产公告，R9智能网联车实现量产。正大100万吨大米加工及1.2万吨米糠油项目取得积极进展。现代服务业发展势头良好。筑石城市会展中心等一批项目运营，优辆车服等本土互联网服务企业快速成长，国内最大的企业外包服务平台“猪八戒网”落户我市。农村电商快速发展，“中国淘宝村”发展到4个，在东北三省占比31%；欧亚商都等商贸企业线上线下销售融合发展。电商网络零售额增长45%以上。科技创新能力增强。产学研用协同创新取得重要进展，中科院苏州医工所吉林市工程技术研究院、吉林大学吉林市研究院挂牌成立，东北电力大学、东杰科技、吉隆科技与邓中翰院士领衔的中星微电子签订战略合作协议。省级以上科技企业孵化器、众创空间达到14个，院士工作站发展到15个。成功获批开展国家创新型城市建设。现代金融支撑有力。银行业金融机构发展到33家，投放信贷资金1000亿元以上。设立4支产业子基金，撬动社会资本35亿元。茜茜家居等10户企业挂牌“吉交所”。人力资源供给不断丰富。深入落实“18条人才新政”，出台集聚人才创新创业实施意见，大学生本地就业率提升到15.2%。顺利通过教育部首批现代学徒试点城市验收，10所中职学校搬迁入驻职教园区，培养技术技能人才8000人。新引进高端领军人才17人、创新团队17个，其中院士7人。

　　（二）三大攻坚战开局良好

　　开展防范化解重大风险攻坚战。坚守不发生区域性系统性风险底线，积极推动吉发集团获得AAA信用等级评定，组建并运行市投融资担保公司，发行我市历史上第一笔境外债，全市未出现一笔债务逾期违约。新增债券资金36.4亿元，置换存量政府债务20.3亿元。加强网络借贷、地方农合机构等风险点监管，立案查处非法集资案件18起。实施断保人员接续缴费奖补措施，积极争取各类支持资金，保障养老金按时足额发放。开展精准脱贫攻坚战。落实“六个精准”要求，开展“携手扶贫”活动和基层基础工作精准化行动，省级贫困村脱贫产业项目实现全覆盖，危房改造、安全饮水和健康扶贫成果显著，42个省级贫困村出列、8594名贫困人口减贫目标如期实现。与白山市扶贫协作取得实效。开展污染防治攻坚战。完成中央环保督察反馈问题整改17项，办结督察转办案件1066件，办结“回头看”转办案件428件。完成省生态环保督察反馈问题整改27项，办结转办案件918件。研究制定了45项常态长效机制。特别是在财政压力较大的情况下，市本级财政追加投入8.2亿元，松花湖生态环境治理、榆树沟社区居民搬迁、“九河一湖”黑臭水体整治等一批困扰我市多年的“老大难”问题启动整改。统筹抓好大气、水、土壤等污染防治，环境空气质量优良天数达到304天，同比增加45天；深入落实“河长制”，松花江干流水质稳定保持在三类良好以上，成为首批国家水生态文明建设试点城市。

　　（三）乡村振兴战略大力实施

　　现代农业发展成效明显。绿色有机水稻种植面积比重提高到37%，实施鑫达农牧科技等重点农业产业化项目14个，省级现代农业产业园发展到6个，市级以上龙头企业增加到318户，“三品一标”农产品达到203个，农产品加工业销售收入增长12%。美丽乡村建设持续推进。巩固“六清”成果，启动农村人居环境整治三年行动，9个精品片区、6个省级美丽乡村、52个省级重点村创建完成。在全省首推农村公路“路长制”，建设和绿美“四好农村路”1358公里。改造农村厕所26050户。明城、岔路河等重点镇污水处理设施竣工运行，农村垃圾收运体系基本实现全覆盖。富农支持力度不断加大。培育新型职业农民3138人，培训各类人员10万人次。建成农村半小时就业服务圈1126个，农村劳动力转移就业61.8万人次。高标准承办全省首届农民丰收节。

　　（四）区域协调发展步伐加快

　　中心城区功能品质提升。建设重点城建工程45项，完成投资51亿元。西部快速路、白山大桥等项目进展顺利，江城广场周边环境改造等12项工程竣工使用。维修更换集中供热老旧管网84.7公里，小街小巷微循环改造25.7万平方米，人民广场等市民生活休闲场所环境提升，城市整体形象进一步改观。县域发展各具特色。磐石市入选全国最具投资潜力百强县并位列全省第一，永吉县被列为全国农村一二三产业融合发展先导区创建单位，舒兰市被命名为中国生态稻米之乡，蛟河市被评为全国中药先进县，桦甸市获评全国首批国民休闲旅游胜地。新型城镇化建设取得进展。大荒地稻香温泉小镇入选国家首批典型经验小镇，明城、烟筒山入选全国千强镇，万昌、平安被评为国家农业产业强镇示范镇，庆岭冰酒小镇等13个小镇列入省级特色产业小镇。

　　（五）重点领域改革深入推进

　　“只跑一次”改革成效显著。创新投资项目审批管理办法，企业投资核准类项目审批时限从165个工作日压减至40个工作日。启动电子政务外网建设，推动政务云平台数据整合，更多业务实现跨部门联办，不动产登记业务办结时限缩短至5个工作日，二手房交易实现2小时内领证。开展无还本续贷业务，为企业节省过桥资金5000万元。建立了以需求管控和清单管理为核心的证明管控体系。率先在全省开展政务大厅“周六轮休、中午不休”延时服务。“只跑一次”改革得到国务院督察组和省政府肯定。国资国企改革不断深化。实施授权经营体制改革，12户市属重点企业超千亿元资产划入吉发集团，国资监管运营体制逐步完善。国有企业42户剥离办社会职能、26户公司制改制、22户“三供一业”资产划转和职能移交任务基本完成。农业农村改革进展顺利。完成农村土地承包经营权确权、国有农场土地确权等重点事项。累计发放土地经营权抵押贷款1.7亿元，土地流转面积占承包地面积的34%，农民合作社、家庭农场分别增长40%和89%。供销合作社综合改革试点经验在全省推广。土地供应机制进一步完善。对经营性房地产用地实行统一规划、统一征收、统一储备、统一供应，全市商品房销售面积同比增长16%，去库存周期同比减少2个月。

　　（六）项目建设和招商引资成效明显

　　扎实开展“招商季”、项目建设推进月等攻坚活动，充分运用投资项目大数据管理平台，强化领导包保和要素保障服务，全年开工5000万元以上项目338项，其中亿元以上项目242项、竣工投产97项。积极参加第三届全球吉商大会、“央企进吉林”等省级经贸交流活动，务实开展与温州对口合作，充分利用正和岛（北大壶）新年论坛等平台，全市各方面积极开展招商活动，蛟河抽水蓄能电站等84个项目签约，吉林建龙60万吨镀锌管等85个项目开工建设，其中京东智慧物流等44个项目实现当年签约、当年开工。

　　（七）民生社会事业持续改善

　　全面完成年初确定的八个方面27件民生实事。城市、农村低保标准分别提高到每人每月545元和每人每年3804元。城市棚户区完成改造4010户，发放住房租赁补贴8555户。560万平方米无籍房办理不动产登记。完成37个老旧小区、1505户农村危房改造任务。新施划一批停车泊位，改扩建停车场53个，静态交通秩序得到有效改善。编制完成新型智慧城市建设总体规划，成为全省首个在公交车上免费提供Wi-Fi服务的城市。全面开展城区小学生课后免费托管服务。基本公共卫生服务项目由12类46项增加到14类55项。大型舞剧《天路》在国家大剧院演出，公共文化服务体系示范区创建通过国家验收。连续3年高水平举办国际马拉松，市城投冰球队参加“丝路杯”VHL超级冰球联赛，城市形象和美誉度大幅提升。民族宗教工作得到加强，圆满承办全省第二届少数民族文艺汇演。军民融合不断深入，双拥共建取得新成效。贯彻落实“四个最严”要求，狠抓食品药品安全监管，有效防控非洲猪瘟疫情；强化安全生产责任落实，大力实施十大领域专项治理；推进信访积案化解攻坚和社会治安综合治理，扎实开展扫黑除恶专项斗争，形成打击遏制的良好态势，有力维护了和谐稳定的社会局面。

　　一年来，全市政府系统认真贯彻全面从严治党部署要求，切实加强党风廉政建设和反腐败工作，深入推进“两学一做”学习教育常态化制度化，扎实开展干部作风大整顿活动，持续加强审计监督，狠抓督查落实，大力促进政府职能转变，政府执行力和公信力不断提升。

　　各位代表，过去一年，我们砥砺前行，奋发作为，取得了极为不易的发展成果，这是以习近平同志为核心的党中央统揽全局、科学决策的结果，是省委省政府和市委坚强领导、凝心聚力的结果，是各县（市）区、各部门和全市广大干部群众拼搏进取、埋头苦干的结果。在此，我代表市政府，向全市各族人民，向人大代表、政协委员、各民主党派、工商联和社会各界人士，向驻吉解放军指战员、武警官兵和公安干警，向中省直各单位以及所有关心、支持吉林市全面振兴发展的海内外朋友，表示衷心的感谢并致以崇高的敬意！

　　在总结成绩的同时，我们也清醒看到，推动我市全面振兴全方位振兴仍然面临不少问题。经济企稳回升的基础还不牢固，各类风险隐患依然存在；新旧动能转换仍需加快，结构性问题还很突出；民生领域还有不少短板，生态环境治理任务仍然艰巨；营商环境与企业和群众的期望还有一定差距，深化改革任重道远。对于这些问题，我们一定以高度负责的精神，认真加以解决。

　　二、2019年工作安排

　　2019年是新中国成立70周年，是全面建成小康社会关键之年。按照市委十三届五次全会部署，政府工作的总体要求是：高举习近平新时代中国特色社会主义思想伟大旗帜，认真贯彻党的十九大、十九届二中、三中全会和中央经济工作会议、全省经济工作会议精神，全面落实习近平总书记对东北振兴工作重要指示精神，统筹推进“五位一体”总体布局，协调推进“四个全面”战略布局，按照全省“三个五”发展战略、中东西“三大板块”建设和“一主、六双”产业空间布局，立足我市“旅游文化名城、新型产业基地、生态宜居城市”的发展定位，坚持稳中求进工作总基调，坚持新发展理念，坚持推动高质量发展，坚持以供给侧结构性改革为主线，坚持深化市场化改革、扩大高水平开放，稳就业、稳金融、稳外贸、稳外资、稳投资、稳预期，打好三大攻坚战，实施乡村振兴战略，推进改革创新，保障和改善民生，奋力开创老工业基地全面振兴全方位振兴新局面。

　　具体说，就是明确一个目标、构建两大格局、打好三大攻坚战、推进六城建设。

　　明确一个目标：

　　地区生产总值增长5—6%，规模工业增加值增长4%，社会消费品零售总额增长6%，一般公共预算收入增长2%左右，单位GDP能耗下降2%以上，城镇和农村常住居民人均可支配收入与经济增长同步。

　　构建两大格局：

　　构建产业发展新格局。近年来，按照“旅游文化名城、新型产业基地、生态宜居城市”的发展定位，坚定不移推进“6411”现代产业体系建设，产业发展的格局更加清晰。旅游依托冰雪资源，以冰雪旅游为先导推进冰雪产业发展已成为全市共识。化工在全国布局调整的大形势下，依托现有发展基础，承接南方化工产业转移优势明显，补链升级、集聚发展的空间仍然很大。新材料产业中碳纤维原丝、碳丝产能快速增长，研发能力不断增强，市场应用空间不断拓展。这三个产业需要我们凝神聚力，快速做优做大做强。

　　构建空间发展新格局。省委省政府做出了构建“一主、六双”产业空间布局的决策部署。我们要主动对接“一主、六双”发展规划，制定我市实施方案，特别要认真分析长吉一体化协同发展给我市带来的机遇和挑战，尽最大可能享受战略实施的红利，成为带动吉林省发展的双核之一。

　　打好三大攻坚战：

　　打好防范化解重大风险攻坚战。积极化解政府债务风险。依法依规盘活财政存量资金，强化政府预算管理，把有限的资金花在刀刃上；规范政府举债行为，拉长置换存量债务期限，新增地方政府债务限额20亿元以上。深化国资国企改革，切实发挥国资监管三级架构核心功能，积极推进平台公司市场化转型。建立国有企业负债约束机制，优化企业债务类型和期限结构，降低流动性风险。有效防范金融风险。搭建重大风险监测预警系统，健全地方金融监管体系，一险一策、分级分类处置农合机构、P2P网络借贷及非法集资等金融风险点，确保不发生系统性区域性金融风险。扎实防控养老保险支付风险。落实振兴东北社会保险惠民援企政策，健全完善参保缴费激励机制，推动基本养老保险参保率达到90%以上，最大化争取中央调剂金支持，确保养老金按时足额发放。加大养老保险政策宣传力度，严格执行政策，严厉打击各种骗保行为。

　　打好精准脱贫攻坚战。全面落实打赢脱贫攻坚战三年行动计划，今年再减少7639名贫困人口、实现21个省级贫困村退出，基本完成脱贫攻坚主要任务。深化与白山市扶贫协作。实施产业可持续化发展工程，推广“携手扶贫”模式，促进脱贫产业提质增效。实施基础设施达标工程，精准规划、精准投入、严格标准，全面完成基础设施建设工作。实施人居环境整治工程，以109个省级贫困村的村庄环境整治为重点，结合农村人居环境整治三年行动，建成一批各具特色的美丽宜居村庄。实施兜底保障工程，加强医疗救助与基本医疗保险、大病保险、低保救助的有效衔接，解决好贫困群体保障问题。实施典型引领工程，坚持扶贫同扶志、扶智相结合，培树一批先进典型，带动更多贫困户自主创业、稳定脱贫。

　　打好污染防治攻坚战。严格按照整改方案和序时进度要求，高质量完成今年任务，确保所有任务在2020年全面完成。对中央环保督察整改剩余的11项反馈问题，必保今年完成5项；尚未办结的26件转办案件和226件“回头看”转办案件，必保今年分别办结14件和137件。对省生态环保督察尚未完成的18项反馈问题，必保今年完成10项；尚未办结的61件转办案件，必保今年办结41件。全力推进重点问题整改，抓好松花湖生态环境治理，坚持分类处置，不搞一刀切；抓好榆树沟社区搬迁，让3382户居民得到妥善安置；抓好黑臭水体整治，推动嘎牙河、北大沟治理工程年底前竣工，城市建成区基本消除黑臭水体。

　　推进六城建设：

　　（一）优化营商环境，着力建设投资兴业之城

　　全面优化营商环境，是推进高质量发展、应对各种复杂变化的重要基础。要着力改革体制机制，降低市场运行成本，积极构建“亲”“清”新型政商关系，助力民营经济大发展快发展，为各类市场主体创造稳定公平透明、可预期的营商环境。

　　1.优化政务环境。组织实施投资项目前期手续和审批服务攻坚大会战，探索推行“标准地+承诺制”新模式。推进政务信息化建设，全面完善政务服务事项“容缺受理”服务机制，无条件开放政务服务e超市。统筹推进“证照分离”和“多证合一”改革，推进电子营业执照跨区域跨部门跨领域应用，逐步实现“一照走天下”。深入推进开发区改革创新，打造营商环境的“制度高地”。着力引进新企业的同时，注重加强对现有企业的服务。

　　2.优化市场环境。实行准入前国民待遇和负面清单制度，大幅降低社会资本市场准入门槛。健全市场综合监管体系，全面建立“双随机、一公开”监管执法体制，实现“阳光监管”。建设信用联合奖惩信息管理系统，设置企业“红名单”，优先向金融机构推介，探索实施失信企业联合惩戒机制。清理政府部门和市属国有企业拖欠民营企业资金，依法依规解决政策承诺不兑现等问题。贯彻全省促进民营经济发展40条意见，研究制定符合我市企业需求、可操作、管用的具体措施，培育“四上”企业80户，民营经济主营业务收入增长7%以上。

　　3.优化法治环境。加强知识产权保护和运用，围绕工业产品、电子商务等重点领域，开展“护航”“雷霆”等执法维权专项行动。搭建法律服务民营企业平台，组建法律服务团20个，开展“律师走访民营企业”活动和“一对一”法律服务。组织开展“平安企业”“平安园区”创建活动，依法严厉打击侵害企业家合法权益、扰乱企业正常经营秩序的违法犯罪行为，倾力打造东北最安全的投资地区。

　　4.优化要素环境。着力破解融资难融资贵问题，整合政府和金融机构资源，搭建产融合作信息服务平台，组织项目发布、银企对接、融资洽谈等活动，信贷投放超千亿元；拓展无还本续贷业务范围，全年无还本续贷额100亿元以上。积极落实国家电价降价政策，帮助50户企业申请列入全省大工业直购电试点范围，全年直购电交易电量力争增长10%。落实减税政策，全年减免企业税负17亿元以上。完善减费措施，坚决取缔无法律法规依据的收费项目，坚决按下限标准收取相关费用，坚决推行建设项目规费征管“一费制”。认真落实国家土地价格政策，对优先发展产业中具备相关条件的项目实行70%价格供地。

　　5.优化社会环境。放宽外来人员落户条件，开辟“服务民营企业绿色通道”，实行民营企业法人凭营业执照、企业职工凭劳动合同和职工名册即可落户政策。对来吉投资、创业、务工人员及引进人才随迁子女，推行入学、升学、转学“一条龙”服务。全面加强卫生健康系统行风建设，继续扩大跨省异地就医直接结算范围。将符合条件的来吉创业、投资人员纳入住房租赁补贴范畴。充分发挥媒体平台舆论宣传主阵地作用，积极弘扬企业家精神，在全社会营造亲商、安商、扶商的浓厚氛围。

　　（二）加快动能转换，着力建设创新发展之城

　　坚持新兴产业培育和传统产业转型升级并重，在稳中有进中完成动能转换，把创新作为动能转换的根本驱动力，推进国家创新型城市建设取得实效。

　　1.突出发展冰雪产业。抢抓政策机遇，打造全国冰雪产业发展集聚区。完善工作机制，成立市冰雪产业发展办公室。推动投资500亿元的中瑞滑雪小镇开工建设，加快实施投资超百亿元的北大壶冰雪体育旅游产业项目，保障北山四季越野滑雪场常年运营，推动万科松花湖滑雪度假区完善功能，在雪场建设上取得新突破；加大冰雪装备制造企业招商引资力度，在永吉经开区规划建设冰雪装备产业园，在引进索道缆车、造雪机、碳纤维滑雪板等冰雪装备制造企业上取得新突破；加强与冬奥组委、国家体育总局、北京延庆区合作，深入实施冰雪奥运冠军培育计划，加强各类场地服务人员培训，承办有影响的冰雪赛事，在服务北京冬奥上取得新突破。深入推动全民上冰雪，浇筑百块室外公益冰场，举办好第四届全市冬季运动会。推动冰雪运动进校园，在全市范围内推广冰雪运动课程，冰雪运动特色校发展到100所。做好“冰雪+”文章，支持北华大学冰雪学院快速壮大，加强与北京体育大学战略合作，加快智慧冰雪创新中心、冰雪科研基地建设，促进冰雪科技研发与成果转化。加大冰雪宣传营销力度，讲好吉林冰雪故事，筹排大型冰雪主题驻场演出，高标准举办雾凇冰雪节等活动，提升冰雪城市品牌影响力。

　　2.着力振兴工业经济。工业是稳增长的压舱石，我市工业项目贷款占全市总量的44%、工业项目投资占总量的43.5%、工业品进出口额占总量的95%。面对稳中有变的经济形势，稳就业、稳金融、稳外贸、稳外资、稳投资、稳预期的任务艰巨，必须把振兴工业作为“六稳”的重要抓手。全力稳定工业运行。落实包保帮扶机制，实施“5640”企业培育计划，发挥骨干企业支撑带动作用。分类制定盘活措施，推进20户以上停产、半停产企业恢复生产，形成一批新的增量。加大要素保障、产需衔接等服务力度，主动帮助企业解决发展难题和历史遗留问题。加快工业转型升级。推动化工“补链”，重点支持吉化公司发展壮大，推动吉化公司炼油结构调整转型升级，确保吉化炼量达到910万吨，力争13万吨丙烯腈等项目落地开工，康乃尔甲醇制烯烃等项目投产运营；依托吉化原料优势，引进江苏苏化精细化学品等一批延长产业链项目。积极推动金珠开发区获批省级开发区。推动冶金“转型”，加快吉林建龙焦化改造、吉林炭素搬迁等项目建设，高标准谋划建设北方小五金生产基地。推动汽车“上量”，全力支持一汽吉林汽车混改，推进圆方机械与一汽富奥并购重组，万丰奥威轮毂达产达效，吉汽龙山汽车零部件产业园开工建设，提升汽车及配套产业能级。推动农产品加工“提质”，加速万昌、孤店子绿色生态农业长廊建设，抓好永吉宇丰米业、桦甸金牛牧业等重点企业发展，加快正大百万头生猪屠宰加工等项目建设，农产品加工业销售收入增长10%以上。推动碳纤维产业壮大并释放产能，建成碳纤维研发中心，推进精功二期、碳纤维拉挤板材等重点项目建设，与中车长客、一汽集团等对接合作，拓展产品在轨道交通车厢、新能源车轻量化、冰雪装备等领域的应用。推动航空、医药健康、先进装备制造业快速发展，实施二台子机场改扩建、康乃尔生物医药产业园、飞机零部件及高端防爆器材等项目，着力培育形成新的经济增长点。加快技术改造和科技创新步伐。围绕产业链部署创新链，瞄准生物、新材料等前沿领域，编制产业技术路线图，建设干热岩协同创新应用中心等创新平台。深入推进校城融合，充分发挥我市高校资源，推动与本地重点产业深度融合。着力提升企业自主创新能力，多渠道增加研发经费投入，实施吉盟2万吨超亮腈纶纤维扩建等技术改造，加快TBM高性能盘形滚刀等产品研发，培育国家高新技术企业20户、“专精特新”企业100户。推动互联网、大数据、人工智能与实体经济深度融合。加快工业数字化改造，推广金洪汽车国家级智能制造试点示范经验，推广吉林建龙冷轧数字化车间等智能制造新模式应用。持续推进国家两化融合管理体系贯标工作，国家贯标评定企业达到5户。抓好制造业服务化，培育瀚丰电气物联平台、东电开元工业互联网平台等一批示范项目。支持各产业领域开展第三方数据分析挖掘服务、技术外包服务，大力发展动漫设计、IT运维、软件外包等数字产业。

　　3.积极发展现代服务业。推动云耕农业、东杰科技等服务业企业与农业、制造业协作联动，加快服务业与一、二产业融合发展。深化全域旅游示范区建设，提升松花湖、雾凇岛等重点景区品牌形象，积极推动以江城美丽山水为背景的特色灯光秀，旅游收入增速保持20%以上。推动欧亚、万达等重点商贸企业提质升级，完善河南街、东市、厦门街等重点商圈服务功能。促进电子商务与快递物流协同发展，推动京东智慧物流园一期、东北亚农产品仓储中心等园区投入运营，网络零售交易额增长30%。

　　4.全力推动“双创”升级。用好人才这个第一资源，紧盯高精尖人才和紧缺型人才，实施人才扶持计划，着力引进一批领军人才和复合型研发团队，强化产学研用协同创新的智力支撑。以创新带动创业和就业，加快建设军民融合等六大创新创业基地，发挥创业就业服务云平台功能，开展项目开发、创业指导、跟踪扶持等“一条龙”“一帮一”服务，发放创业担保贷款1.7亿元。弘扬劳模精神和“工匠精神”，举办创新创业大赛，倡导崇尚创新、宽容失败的创新文化，激发全社会创新创业活力。

　　（三）统筹区域布局，着力建设协调发展之城

　　坚持点线面结合，既尊重特色，实施错位发展；又注重协同，实施联动发展，形成城乡、区域、县域之间协调发展的合力。

　　1.实施乡村振兴战略。坚持质量兴农。落实粮食安全省长责任制，新增绿色有机水稻面积2000公顷，提前一年完成“十三五”高标准农田建设任务。巩固中国粳稻贡米之乡品牌优势，积极推介农产品区域公用品牌。坚持产业强农。推广“公司+基地+合作社+农场+农户”发展模式，建设10个产业基地、5户产业化联合体示范企业。加快设施农业发展，推动棚膜生产由单季向四季转变。加快发展休闲农业，市级以上星级企业发展到120户。坚持基础支农。清理整治“大棚房”问题，坚决遏制农地非农化乱象。实施农田水利设施建设58项，建设和绿美“四好农村路”1500公里，推广应用先进适用技术20项以上。坚持改革助农。启用农村土地确权信息应用平台，实现市县乡三级互联互通。引导各类主体发展适度规模经营，农村土地流转面积保持14万公顷以上。深化农村产权制度、农垦、供销社等方面改革。坚持增收富农。实施能人回乡、工商兴乡、社会助乡“三乡”工程，培育新型职业农民2500人，农村劳动力转移就业55万人次。建设丰满温德河四季生态等一批田园综合体，让更多农民充分参与和受益。

　　2.推动长吉一体化协同发展。认真抓好“一主、六双”产业空间布局在我市的规划实施，找准定位，明确任务。要加快长吉都市圈建设，开展化工与汽车、碳纤维与轨道客车等产业战略对接，实施二氧化碳基聚醚多元醇、一汽吉林C023等项目，为我市化工、碳纤维、旅游等产业拓展市场空间。率先推动交通路网联通，规划建设旅游市域轨道交通，推进国道珲乌公路吉林至饮马河段一级公路等重大项目，提升长吉南线、长吉北线等旅游、运输大通道通行质量，增强连接聚合能力。强化节点支撑，推动永吉与城区同城化发展，抓好桦皮厂、岔路河等长吉重要节点镇建设，实现产业和人口集聚。

　　3.促进县域经济发展。推动资源依赖型经济转型。抓好金属矿产等“原字号”产品的精深加工和产业链延伸，积极培育全域旅游、电子商务等新兴产业和现代服务业，努力走出一条资源转化、产业转型、发展方式转变的新路。推动产业园区建设。整合优化布局，培育永吉汽车配套生产、舒兰钼资源开发、磐石新材料、蛟河医药健康、桦甸新能源开发等产业集群，构建优势互补、错位竞争、协同发展的区域经济格局。推动县域晋位升级。促进县域经济总量壮大、后劲增强、综合实力提升，力争各县（市）在国家和省综合实力排名位次前移。

　　（四）坚持生态优先，着力建设绿色发展之城

　　绿水青山是我市的发展优势。要牢固树立绿水青山就是金山银山的理念，进一步增强绿色发展的自觉性和主动性，不要以牺牲环境为代价的发展，保护好天蓝地绿水清的美丽家园。

　　1.巩固提升绿色发展优势。打好蓝天保卫战。深入开展工业污染防治和煤烟型污染防治，基本淘汰县级城市建成区10蒸吨及以下燃煤小锅炉。全面开展秸秆露天禁烧专项行动，抓好秸秆禁烧区、限烧区管控，提高综合利用水平。严格控制煤炭消费总量，开展散煤治理和煤炭减量替代等工作。全年环境空气质量优良天数保持80%以上。打好碧水保卫战。全面落实“河长制”，开展好河湖“清四乱”专项行动。推进县级以上集中式水源地规范化建设，实施水源地安全保障达标工程。推动蛟河天岗等工业集中区污水处理厂竣工运营，地表水国控断面一至三类水体比例达到83%以上。打好青山、黑土地保卫战。持续开展“绿盾”自然保护区监督检查专项行动，严肃查处保护区内违法违规开发行为。加快农业面源污染治理，保护和利用好黑土地资源。开展重点行业企业用地土壤污染状况调查，严格限制高风险化学品生产使用，严厉打击固体废物环境污染违法犯罪行为。打好环境安全保卫战。修订非煤矿山尾矿库环境应急预案，加强危化品运输管控和应急设施建设，完善松花江吉林市江段22个水污染防控断面应急防控措施。深化生态环境损害赔偿制度改革，落实生态保护红线划定政策，实施山水林田湖生态修复工程，带动生态环境质量整体改善。

　　2.大力推动产业绿色发展。加快发展水电、生物质发电、风电等清洁能源，抓好丰满大坝治理、蛟河抽水蓄能电站等项目建设，清洁能源供应量增长4%。支持金隅冀东环保科技等企业创建“绿色工厂”，实施吉林化纤、瀚禹化工等企业绿色制造项目建设。严格执行建筑节能标准和绿色建筑要求，绿色建筑占新建建筑比例提升到40%以上。推进博大生化等企业完成清洁生产技术改造，降低污染物排放强度。建立完善推动落后产能退出工作机制，依法依规淘汰落后产能。

　　（五）扩大合作交流，着力建设开放发展之城

　　扩大对外交流合作，引进项目、资金、技术、管理，形成新的经济增长点，是我市发展的必然选择。按照全省“项目建设年”部署，把项目建设和招商引资作为振兴发展的关键所在，充分发挥开发区主战场作用，举全市之力，确保新开工工业项目占地5平方公里以上，投资强度达到每平方米3000元。全年开工5000万元以上重点项目330项，其中，亿元以上项目245项、10亿元以上项目40项；“三早”项目190项。

　　1.开展全方位对口合作。紧紧把握我省与浙江省对口合作机遇，利用好与温州对口合作机制，推进与温州各县（市）区、开发区合作，提高两地产业融合发展水平。持续开展“浙商吉林行”等活动，引导温商、浙商资本更多进入吉林市场，促进两地资本流动、资源共享。深入开展吉林优质产品温州系列宣传推介活动，进一步提高在温州、浙江市场的品牌影响力。支持两地开展中高端人才互认共享，为对口合作提供坚实的智力支持。

　　2.开展多层次战略合作。主动融入全省“1+N+X”战略合作框架，全面对接京津冀、长江经济带、粤港澳大湾区和东北四省区产业协作需求，务实开展化工、冰雪、新材料、生物制药等专题专项招商活动。加强城市间互动交流，推动与桂林、苏州、厦门等重点旅游城市实现宣传互动、资源共享、游客互换。创新开发区合作发展，开展“飞地经济”试点，与大亚湾等产业相近开发区建立交流合作机制。提升域内企业对外合作水平，推动吉化ABS专用料在更多白色家电企业供应使用，推进小糸车灯与华晨宝马、北京现代实现产品配套。

　　3.开展紧密型专业合作。深化与中科院等科研院所合作，支持已落户的院士工作站发挥更大作用，打造苏州医工所吉林市工程技术研究院等科研合作示范典型。促进浙江大学二次技术转移项目落地，深化市中心医院、吉林医药学院与温州医科大学的专业合作，推进市人民医院与北医大三院共建运动医学中心，加快吉林大学吉林市研究院建设，科技成果就地转化20项以上。密切与河北化工、江苏医药、浙江汽车等商企协会对接合作，再建立友好商会5个以上。积极参加科研院所、著名高校、知名商会进吉林等活动，再形成一批重大合作成果。

　　4.开展前瞻性共建合作。抢抓国家支持东北振兴机遇，围绕基础设施建设、生态环保、乡村振兴、民生工程等领域，谋划梳理重大项目160项，强化项目包装，加大“上争”力度，争取轨道交通等更多项目纳入国家计划。依托国家创新型城市、国家产融合作试点城市、国家电子商务与物流快递协同发展试点城市等优势，用好用足国家部委政策，积极开展先行先试，最大限度加快振兴发展步伐。

　　5.开展开放性国际合作。推动与瑞士巴涅市、法国纪尧姆市等世界冰雪名城建立友好关系，拓宽与俄罗斯伏尔加格勒市、日本松江市等国际友城的合作领域，将与东南亚等重点地区合作引向深入。加快中新吉林食品区发展步伐，深化与泰国正大集团等大企业的合作，打造国家农业高新技术产业示范区，建成东北亚开放合作的重要平台。完善保税物流中心功能，扩区增项，拓展跨境电商业务，打造全省外经贸发展新窗口。依托吉林创新科技城、吉林软件服务外包基地等载体，吸引品牌服务贸易企业集聚发展，积极创建全省服务出口基地。培育吉林炭素超高功率石墨电极等高附加值出口产品，助力开拓俄罗斯等国外市场。

　　（六）切实保障民生，着力建设共享发展之城

　　坚持以人民为中心发展思想，尽力而为、量力而行，兜底线、保基本、提品质，不断增强人民群众的获得感、幸福感和安全感。

　　1.扎实办好民生实事。今年要办好七个方面30件民生实事。促进社会就业。深入落实国家、省、市各项促进就业政策，城镇新增就业6.8万人，城镇登记失业率控制在4.5%以内。完善社会保障。农村低保标准不低于当年度国家现行扶贫标准，城市低保标准不低于上年度人均可支配收入的20%。对低保家庭中的重病患者、重残人员、老年人、未成年人按平均不低于50%低保标准的比例增发补助金。实施安居工程。坚持实物保障与租赁补贴并举，发放住房租赁补贴8000户。改造城市棚户区556户，解决2142户居民棚改逾期未安置问题。存量无籍房不动产登记任务完成70%以上。改造老旧小区达到50%以上。推进“一城一网”和热源能力提升联网工程建设，改造供热老旧管网53公里。建立物业服务企业信用信息体系，规范物业服务市场，提升物业服务水平。改造农村厕所40720户。发展教育事业。落实学生资助专项资金，不让一名孩子因贫失学。深化大学区管理改革，促进义务教育优质均衡发展。筹建吉林通用航空高等职业技术学院，促进职教园区高效运转，提高现代职业教育发展水平。完善健康服务体系。将城镇居民基本医疗保险困难群众个人缴费纳入财政补助范畴。低保困难家庭患者核磁共振、CT检查费减免70%。免费为1万名贫困人口开展消化道胃癌早期筛查治疗。免费向3万名在校中小学生提供视力筛查服务。持续开展养老院服务质量提升专项行动，医养结合率达到90%以上，二级以上医疗机构开通老年人就医绿色通道。实施“畅通吉林”工程。绕城高速向城市7座及以下小客车免费开放。打通西山街等4条断头路。对19条次干路、150条支路、200条胡同路面进行集中治理，畅通群众出行“最后一公里”。加强静态交通管理，全面开展核心区静态交通治理，启用中心医院周边区域静态交通设施，改造升级28处道路停车场，实施机动车停放网格化管理，有效缓解交通拥堵压力。实施文体惠民工程。推进国家引导城乡居民扩大文化消费试点城市建设，举办庆祝新中国成立70周年国乐艺术周等系列文化活动，推进非物质文化遗产展示馆建设，传承和弘扬优秀传统文化，丰富城乡文化生活。高水平举办国际冰雪摄影大展、第四届国际马拉松、中芬冬季运动年等赛事活动，进一步提升城市知名度。

　　2.提升城市建设管理水平。实施50项城建重点工程，当年完成投资60亿元以上。推进智慧城市建设。统筹实施雪亮工程、智慧化停车和重点区域免费Wi-Fi覆盖等城市信息化建设。启动居民“一卡通”项目，实现社会保障、城市交通等公共服务功能一卡通办。统一整合全市各类政务服务和便民服务热线，构建“12345”一号通便民服务热线平台。推进海绵城市建设。启动实施哈达湾区域22条道路、海绵型公园等海绵城市建设试点。建设功能完善的监测平台，实现海绵城市建设动态情况的数据采集和远程传输。推进文明城市建设。以创建全国文明城市为契机，全面实施“九大工程”，开展“九大行动”，健全公共环境、公共秩序、交通和社区文明行为规范，扎实推进“温暖家庭、幸福和谐社区”建设。集中拆除2600处侵占消防通道等违建设施，治理小区野广告张贴行为，打造吉林一中、毓文中学等35所校园周边环境示范区，建设干净、美丽、安全、有序的文明江城，促进城市公共文明指数和群众满意度“双提升”。

　　3.持续巩固社会安全稳定局面。深入落实“四个最严”要求，健全监管体系，切实保障公众饮食用药安全。全面落实全省党政领导干部安全生产责任制实施细则，全面完善安全生产制度机制，全面加强应急救援能力建设，坚决遏制重特大事故发生。进一步做好民族宗教工作。加大“事要解决”力度，妥善处理好群众的合理诉求。深入开展扫黑除恶专项斗争，实现深挖根治的阶段性目标，全面推进平安江城建设。

　　各位代表，新时代要有新气象、新担当、新作为！

　　我们要提高政治站位，树牢“四个意识”，坚定“四个自信”，坚决做到“两个维护”，始终在政治立场、政治方向、政治原则、政治道路上同以习近平同志为核心的党中央保持高度一致。

　　我们要依法全面履职，自觉接受市人大及其常委会的法律监督、工作监督和市政协的民主监督，虚心接受社会监督和舆论监督，平稳完成机构改革，使政府各项工作始终在法治轨道上运行。

　　我们要切实转变作风，坚决落实中央八项规定精神，深入推进“两学一做”学习教育常态化制度化，认真开展“不忘初心、牢记使命”主题教育，驰而不息纠正“四风”，解放思想，奋发作为，以昂扬向上的精神状态狠抓落实、开拓事业。

　　我们要落实全面从严治党要求，强化反腐倡廉，规范权力运行，始终把纪律和规矩挺在前面，努力打造一支忠诚、干净、担当的干部队伍。

　　各位代表，新思想引领新时代，新使命开启新征程。让我们高举习近平新时代中国特色社会主义思想伟大旗帜，在省委、省政府和市委的坚强领导下，深入贯彻落实习近平总书记对东北振兴工作重要指示精神，不忘初心、牢记使命，锐意进取、埋头苦干，为实现新时代吉林市全面振兴全方位振兴而努力奋斗！
